# Supplementary material for: EnzML: multi-label prediction of enzyme classes using InterPro signatures
Source: BMC Bioinformatics. 2012 Apr 25;13:61. doi: 10.1186/1471-2105-13-61 (PMC3483700; doi:10.1186/1471-2105-13-61)
Supplement: Addtional file 5 — The Java code to format the data files, evaluate and predict. The file enzml_java_code.tar.gz contains the Java code used to format database data to ARFF and XML formats, to execute cross and train-test (jackknife) evaluations and to record evaluation results to database. More information is included in the readme.txt file and the Javadoc files. The code can be used with a MySQL database. To use a different database software, other JDBC drivers might be required. [file 1471-2105-13-61-S5.gz › java_code/utils/doc/uk/ac/ed/inf/utils/webutils/simpledomparser/class-use/XmlNode.html]

Uses of Class uk.ac.ed.inf.utils.webutils.simpledomparser.XmlNode


---


|  |  |  |
| --- | --- | --- |
| **Overview** \| **Package** \| **Class** \| **Use** \| **Tree** \| **Deprecated** \| **Index** \| **Help** | |  |
| PREV   NEXT | **FRAMES**    **NO FRAMES**     **All Classes** |  |


---


## **Uses of Class uk.ac.ed.inf.utils.webutils.simpledomparser.XmlNode**

| Packages that use XmlNode | |
| --- | --- |
| **test** |  |
| **uk.ac.ed.inf.utils** |  |
| **uk.ac.ed.inf.utils.webutils** |  |
| **uk.ac.ed.inf.utils.webutils.simpledomparser** |  |

Uses of XmlNode in test

| Methods in test that return XmlNode | |
| --- | --- |
| `static XmlNode` | `XmlNodeTest.getNumberedXmlTree()` |
| `static XmlNode` | `XmlNodeTest.getPdbSimpleElement()` |
| `static XmlNode` | `SimpleDOMParserTest.parseAndGetSimpleElementTest()` |

Uses of XmlNode in uk.ac.ed.inf.utils

| Methods in uk.ac.ed.inf.utils that return XmlNode | |
| --- | --- |
| `static XmlNode` | `EntrezUtils.getESummaryXML(java.lang.String db, java.lang.String id)`             Gets an XML tree of elements (SimpleElement) from eSummary Entrez service. |

| Methods in uk.ac.ed.inf.utils with parameters of type XmlNode | |
| --- | --- |
| `static java.lang.String` | `EntrezUtils.eSummaryToString(XmlNode xmlTree)`             Test method to print an an Entrez eSummary xml report |
| `static java.lang.String` | `EntrezUtils.xmlElementToString(XmlNode xml)`             Test method to print to string a single Entrez eSummary xml element |

Uses of XmlNode in uk.ac.ed.inf.utils.webutils

| Methods in uk.ac.ed.inf.utils.webutils that return XmlNode | |
| --- | --- |
| `static XmlNode` | `XMLUtils.getXmlFromHTML(java.lang.String url, java.lang.String xhtmlFile)` |
| `static XmlNode` | `XMLUtils.getXmlFromReader(java.io.BufferedReader reader)`             Get the XML as SimpleElement from a URL reader. |

Uses of XmlNode in uk.ac.ed.inf.utils.webutils.simpledomparser

| Methods in uk.ac.ed.inf.utils.webutils.simpledomparser that return XmlNode | |
| --- | --- |
| `XmlNode` | `XmlNode.addChildNode(XmlNode element)`             Adds a child element to the node, and returns the node itself |
| `static XmlNode` | `XmlSearcher.depthFirstSearch(XmlNode xmlTree, java.lang.String searchString, java.lang.String attributeName, java.io.File log)`             Searches this tree in a depth first mode and returns the first node whose tag, text or attribute value matches the given string. |
| `static XmlNode` | `XmlSearcher.depthFirstSearch(XmlNode xmlTree, java.lang.String searchString, java.lang.String attributeName, java.io.File logFile, int emergencyBrake, int maxNumberOfChildren)`             Searches this tree in a depth first mode and returns the first node whose tag, text or attribute value matches the given string. |
| `static XmlNode` | `XmlSearcher.depthFirstSearchOfParent(XmlNode tree, java.lang.String desiredTag, java.lang.String attributeName)` |
| `static XmlNode` | `XmlSearcher.depthFirstSearchOfParent(XmlNode xmlTree, java.lang.String searchString, java.lang.String attributeName, java.io.File logFile, int emergencyBrake, int maxNumberOfChildren)`             TODO duplicated code! depthFirstSearch ~ depthFirstSearchOfParent Searches this tree in a depth first mode and returns the \*parent\* of the first node whose tag, text or attribute value matches the given string. |
| `static XmlNode` | `XmlNode.getXmlTreeFromString(java.lang.String XMLText)` |
| `static XmlNode` | `XmlNode.getXmlTreeFromUrl(java.lang.String url, boolean gzipped)`             get XML tree from URL |
| `XmlNode` | `SimpleDOMParser.parse(java.io.Reader reader)` |
| `XmlNode` | `XmlNode.removeChildNode(XmlNode node)`             Remove a child element |

| Methods in uk.ac.ed.inf.utils.webutils.simpledomparser that return types with arguments of type XmlNode | |
| --- | --- |
| `java.util.LinkedList<XmlNode>` | `XmlNode.getChildElements()`             public Object[] getChildElements() { return m\_childElements.toArray(); } |
| `static java.util.LinkedList<XmlNode>` | `XmlSearcher.getInternalBlocks(java.lang.String externalBlockTag, XmlNode xmlTree, java.io.File log)`             Gets the children of a given xml tag |

| Methods in uk.ac.ed.inf.utils.webutils.simpledomparser with parameters of type XmlNode | |
| --- | --- |
| `XmlNode` | `XmlNode.addChildNode(XmlNode element)`             Adds a child element to the node, and returns the node itself |
| `static XmlNode` | `XmlSearcher.depthFirstSearch(XmlNode xmlTree, java.lang.String searchString, java.lang.String attributeName, java.io.File log)`             Searches this tree in a depth first mode and returns the first node whose tag, text or attribute value matches the given string. |
| `static XmlNode` | `XmlSearcher.depthFirstSearch(XmlNode xmlTree, java.lang.String searchString, java.lang.String attributeName, java.io.File logFile, int emergencyBrake, int maxNumberOfChildren)`             Searches this tree in a depth first mode and returns the first node whose tag, text or attribute value matches the given string. |
| `static XmlNode` | `XmlSearcher.depthFirstSearchOfParent(XmlNode tree, java.lang.String desiredTag, java.lang.String attributeName)` |
| `static XmlNode` | `XmlSearcher.depthFirstSearchOfParent(XmlNode xmlTree, java.lang.String searchString, java.lang.String attributeName, java.io.File logFile, int emergencyBrake, int maxNumberOfChildren)`             TODO duplicated code! depthFirstSearch ~ depthFirstSearchOfParent Searches this tree in a depth first mode and returns the \*parent\* of the first node whose tag, text or attribute value matches the given string. |
| `static boolean` | `XmlSearcher.evaluateCondition(XmlNode element, java.lang.String wantedString)`             Check whether the desired string is either a tag or text for this element. |
| `static boolean` | `XmlSearcher.evaluateCondition(XmlNode element, java.lang.String wantedString, java.lang.String attributeName)`             Check whether the desired string is either a tag, text or an attribute \*value\* for this element. |
| `static java.util.LinkedList<XmlNode>` | `XmlSearcher.getInternalBlocks(java.lang.String externalBlockTag, XmlNode xmlTree, java.io.File log)`             Gets the children of a given xml tag |
| `static java.lang.String` | `XmlSearcher.getTextFromChildElement(XmlNode xmlTree, java.lang.String searchString, java.lang.String attributeName, java.io.File log)`             Checks if child exists and returns its text |
| `XmlNode` | `XmlNode.removeChildNode(XmlNode node)`             Remove a child element |

---


|  |  |  |
| --- | --- | --- |
| **Overview** \| **Package** \| **Class** \| **Use** \| **Tree** \| **Deprecated** \| **Index** \| **Help** | |  |
| PREV   NEXT | **FRAMES**    **NO FRAMES**     **All Classes** |  |


---
